# High Biodiversity on a Deep-Water Reef in the Eastern Fram Strait

**DOI:** 10.1371/journal.pone.0105424

**Published:** 2014-08-25

**Authors:** Kirstin S. Meyer, Thomas Soltwedel, Melanie Bergmann

**Affiliations:** 1 Oregon Institute of Marine Biology, Charleston, Oregon, United States of America; 2 Alfred-Wegener-Institut Helmholtz-Zentrum für Polar- und Meeresforschung, Bremerhaven, Germany; Université du Québec à Rimouski, Canada

## Abstract

We report on the distribution and abundance of megafauna on a deep-water rocky reef (1796–2373 m) in the Fram Strait, west of Svalbard. Biodiversity and population density are high, with a maximum average of 26.7±0.9 species m^−2^ and 418.1±49.6 individuals m^−2^ on the east side of the reef summit. These figures contrast with the surrounding abyssal plain fauna, with an average of only 18.1±1.4 species and 29.4±4.3 individuals m^−2^ (mean ± standard error). The east side of the reef summit, where the highest richness and density of fauna are found, faces into the predominant bottom current, which likely increases in speed to the summit and serves as a source of particulate food for the numerous suspension feeders present there. We conclude that the observed faunal distribution patterns could be the result of hydrodynamic patterns and food availability above and around the reef. To our knowledge, this study is the first to describe the distribution and diversity of benthic fauna on a rocky reef in deep water.

## Introduction

The deep sea is generally characterized by soft sediments, with hard-bottom habitats representing anomalies, though hard-bottom habitats are often home to a variety of species and functional groups not found elsewhere [1]. Bathymetry surveys at the deep-sea observatory HAUSGARTEN in the eastern Fram Strait revealed an anomalous feature on the slope west of Svalbard, consisting of a depression in the continental slope adjacent to a steep crescent-shaped rocky reef. A reef is here defined as a 3-dimensional, hard-bottom structure which provides habitat for sessile organisms and fish. Rising over 500 m depth in just 800 m horizontal distance, the present deep-water reef features sheer, rocky faces covered by a wide variety of sponges, anemones, and soft corals.

Rocky reefs are relatively well-known from shallow water, especially in terms of their fish fauna. Research has centered on how habitat structure affects the distribution [2], [3], [4], [5], [6], [7] and recruitment [8], [9], [10] of fish fauna on coral and rocky reefs, the impacts of fish predation on rocky reef communities [11], [12], as well as the implications of marine reserves and artificial reefs [13], [14] for conservation and fisheries management. Sessile invertebrate fauna have also been described for subtidal rocky shores [15], [16], [17], [18], oyster reefs [19], rocky reefs [20], deepwater coral reefs [21] and artificial reefs [14]. To our knowledge, the present study is the first to describe the distribution and abundance of fauna on a rocky reef in the deep sea.

Because the global deep seafloor is largely characterized by soft, organically-derived sediments, any structure of hard substratum presents an anomaly. Isolated hard structures such as manganese nodules [22], dropstones [23], [24], and sea urchin tests [25] provide habitat islands for sessile organisms. Coldwater corals and hexactinellid sponges are found associated with hard surfaces on the walls of submarine canyons and fjords [26], [27], [28], [29]. Seamounts, which may feature exposed bedrock, are well-known as unique structures in the deep sea which support large stands of suspension-feeding organisms, particularly cold-water corals that take advantage of increased current speed and altered circulation patterns over the top of the seamount [30], [31].

The objective of the present study is to describe the distribution, abundance, and diversity of sessile invertebrate fauna found on a deep-water rocky reef in the Fram Strait. Throughout the discussion, the majority of taxa observed on the reef will be referred to by pseudonyms ( = morphospecies), as not all have yet been clearly taxonomically identified or described. It is suspected that a large number of species present at this station are new to science (D. Janussen, pers. comm., 2012). The collection of sessile organisms from hard substrata requires the use of a work-class Remotely Operated Vehicle (ROV) for targeted sampling. Since the study site was covered by sea ice during a planned dive in 2013, ground-truthing could not be realized to date. Nevertheless, as this paper is intended as an ecological rather than a taxonomic treatment of the fauna, we believe that the important patterns in the ecological community are able to be sufficiently discerned by the identification of morphospecies, as has been shown in other benthic environments [32].

## Methods

### Study location and image collection

**Figure 1:**
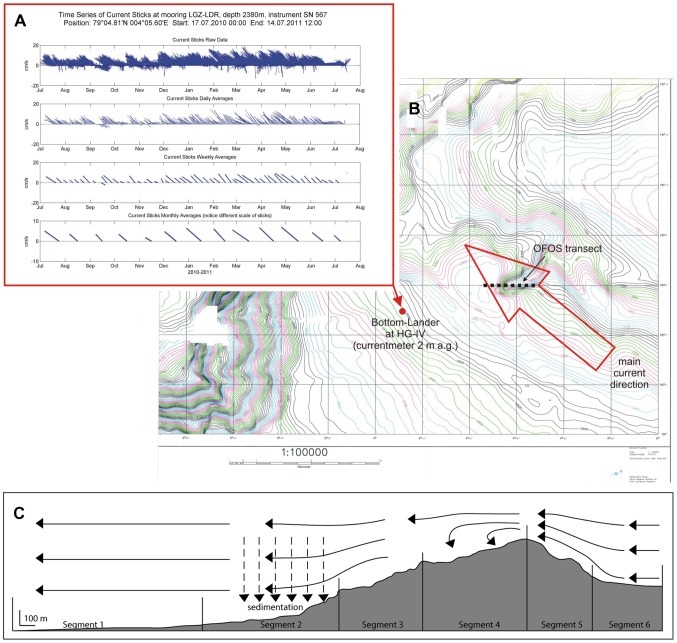
Physical environment of the deep-water rocky reef. A, velocity of the bottom current as measured by a long-term lander at station HG-IV; B, bathymetry of the deep reef with location of the photographic transect and predominant bottom current; C, topography of the reef showing transect segments and presumed hydrodynamic patterns over the reef.

On July 27, 2012, images were recorded from a photo transect along a deep reef using a towed underwater camera system. The reef is located in the eastern Fram Strait, west of Svalbard at approximately 79° 06′ N/04° 28′ E, and it lies within the HAUSGARTEN oceanographic observatory [33]. The photo transect was begun at 79° 05.98′ N/04° 23.01′ E (2332 m depth), and ran due east across the reef summit (1796 m) to end at 79° 06.02′ N/04° 33.92′ E (2084 m) giving a total transect length of 3.82 km (Fig. 1). The HAUSGARTEN observatory is not privately owned or protected, and it is located in international waters. Therefore, no specific permits were required for collection of data. To our knowledge, no species observed in this study are endangered, and no negative impact on the biota was made during collection of photographic data. The camera system used in this study, the Ocean Floor Observation System (OFOS), consists of a vertically-facing camera, flashes, and three red laser points for size reference. Images were recorded automatically every 30 seconds, and additional manually-triggered images were also recorded when an object of particular interest occurred in the camera's field of view. The target camera altitude was 1.5 m. Assuming a constant speed of 0.5 knots, automatically-triggered images were spaced approximately 8 m apart horizontally. Additional details were described by Meyer et al. [34] for 2012 photographic sampling.

### Image labeling

Image analysis was conducted using the web-based image analysis program and database BIIGLE (Bio-Image Indexing, Graphic Labeling, and Exploration; www.biigle.de) [35], [36]. Three laser points present in each image were detected by a computer algorithm and used as a standard to calculate the camera footprint, which could then be used to convert taxon counts to densities. The ship's motion during OFOS deployment caused altitude to vary slightly between images. To reduce variability in densities/diversity estimates due to differences in camera altitude, we restricted our analysis to images taken between 1.3 and 1.6 m altitude. Because of a shortage of usable automatically-recorded images from segment 5 (see below) of the transect, two automatically-recorded images of altitude 1.7 and 1.8 m, as well as 9 manually-recorded images of altitude 1.3–1.5 m, were included in the analysis of this segment. Image labeling was completed in a shaded room using a 20”computer monitor connected to a PC. All images were investigated using the maximum available zoom in BIIGLE (version 2013), and all observable biota and biotic habitat features were labeled. Habitat features included organism tracks in the soft sediment (“Lebensspuren”), debris of dead *Caulophacus arcticus*, crinoid stalks, burrow entrances, shell fragments, worm tubes, and an unidentifiable structure termed “hairball.” To eliminate practice effects, each image was examined twice, one time each on two different days. Image analysis was completed by the same individual (KSM) to avoid intra-observer variability [37].

### Statistical analysis

Percent hard-substratum cover was estimated for each image by overlaying a grid of 90 regularly-spaced points and observing how many points met hard substratum or soft sediment. For statistical analysis, the transect was divided into six segments, the edges of which were marked by topographic breaking points of the reef (Fig. 1C). Each breaking point corresponded to a change in the slope of the reef as well as a noticeable difference in hard-substratum cover. Splitting the transect in this manner allowed the abyssal plain community above and below the reef, which was photographed as part of the same transect, to be analyzed separately and compared to the reef community. Also, different sections of the reef with different topographical characteristics (slope, substratum, facing into or away from predominant current) could be compared to one another. The first 15 randomly-selected images from each transect segment were used for analysis, and these images were treated as replicate samples within their respective transect segments.

Diversity indices including Margalef's richness [38], Pielou's evenness [39], and Shannon-Wiener diversity [40] were calculated for each image using Primer v6 [41]. Densities of biota and habitat features were compared between transect segments using (non-) parametric analyses of variance in SPSS (IBM, USA). A Levene's test was used to test homogeneity of variance. In the instance that a log(x+1)-transformation ensured equal variance, an ANOVA test on log(x+1)-transformed data was used, and post-hoc Bonferroni tests indicated pairwise differences. For cases of unequal variance, a Kruskal-Wallis test was used, and pairwise differences between the years were discerned using Mann-Whitney *U* tests with a Bonferroni correction of p = 0.05/15 comparisons  = 0.003. Non-parametric Spearman correlations were also conducted in SPSS, and multivariate statistics including ANOSIM, MDS, and SIMPER were conducted using fourth root-transformed data in Primer v6 [41].

## Results

### Description of the abiotic environment

The reef observed in this study appears to be an outcropping of the continental slope west of Svalbard. Flanked by soft-sediment environments on three sides, the reef itself spans a depth range of 576 m (1796–2373 m). It is oriented in a generally southwest-to-northeast direction, and it lies adjacent to a depression in the seafloor approximately 3.5 km in diameter. Current-meter data from a long-term mooring at the nearby central HAUSGARTEN station HG-IV [33] indicate the predominant near-bottom current direction in the area is to the northwest at approximately 5 cm s^−1^ (Fig. 1A,B). Therefore, the ridge formed by the summit of the reef lies approximately perpendicular to the predominant current. The bottom current flows along segment 6 and increases in speed because the same volume of water passes through a smaller area as the depth decreases. On segment 5, the bottom current is fast enough to reduce sedimentation or even erode loose sediment, as evidenced by exposed bare rock observed in the images. Significant turbulence is likely present at the summit and along segments 4 and 3, and this may increase vertical mixing. Sedimentation is likely to occur as current slows along segments 2 and 1.

**Figure 2:**
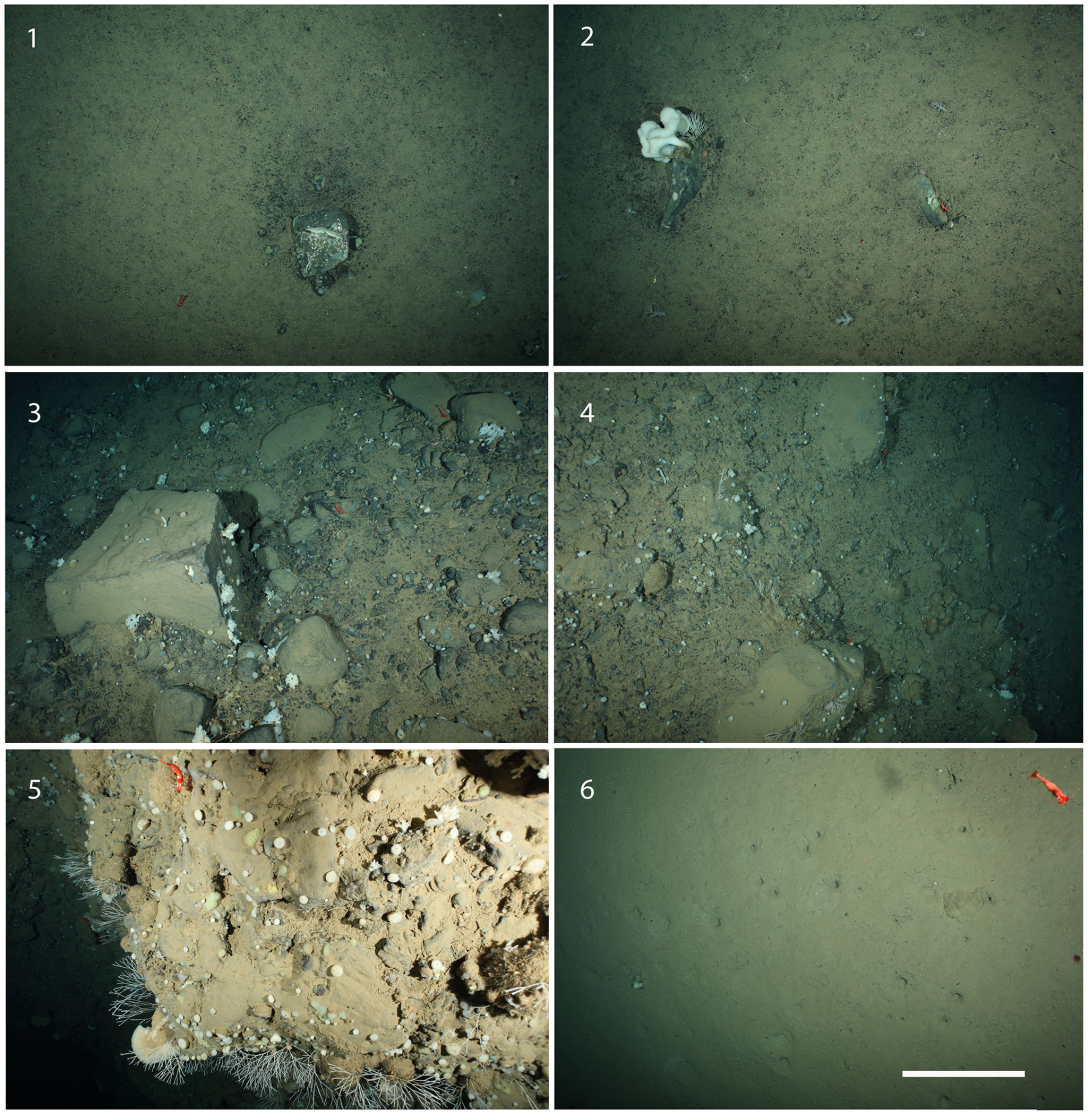
Typical images from each transect segment showing substrata and fauna.

The OFOS transect runs west to east over the reef (Fig. 1B); therefore, fauna were observed in a direction opposite to the main current. Segment 1 is characterized by soft sediment with occasional dropstones (isolated stones, which were most likely released from melting icefloes [23], [24]) at a mean density of 0.8 m^−2^, resulting in an average 14.8% hard-substratum cover. Segment 1 is relatively flat, sloping upward at only 1.8°. Segment 2 has an average of 28.0% hard-substratum cover and a 12.2° slope, while segment 3 has an average of 61.1% hard-substratum cover and a 17.7° slope. Segment 4 is located at the summit of the reef on the west (leeward) side; it is characterized by an average of 54.5% hard-substratum cover and a 13.7° slope. On the opposite side of the summit facing the east, segment 5 has a high occurrence of bare rock that indicates erosion of loose sediment; it is characterized by an average of 91.2% hard-substratum cover and a 29.4° slope. At the end of the transect, segment 6 is once again characterized by soft sediment with an average of 7.9% hard-substratum cover and a 6.5° slope. Figure 2 shows a typical image from each transect segment.

### Distributions of taxa

Altogether, 65 morphospecies were observed in the images. Of these, five were excluded because of ambiguity in their identification; the remaining 60 were used for analysis. Each morphospecies is believed to constitute only one species based on observed morphological characteristics. In some cases, species were able to be identified by comparison to images and voucher specimens collected from nearby stations in the HAUSGARTEN observatory; however, the majority of morphospecies cannot yet be properly identified without collection of specimens from the reef itself. Most morphospecies will thus be referred to here by pseudonyms. Morphospecies and species will be collectively referred to as “taxa.”

**Figure 3:**
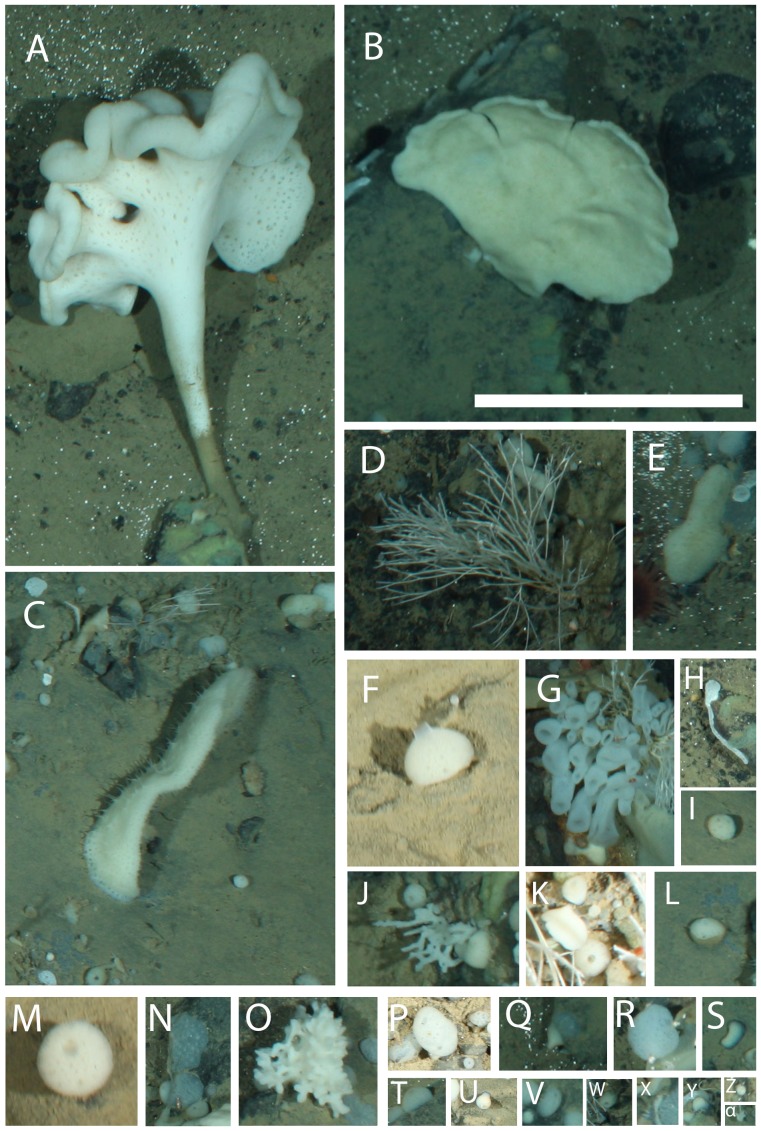
Sponges observed at the deep reef. A, *Caulophacus arcticus*; B, narrow white sponge; C, hairy white sponge; D, *Cladorhiza gelida*; E, puffy white encrustment; F, *Polymastia*; G, cup sponge; H, thin white encrustment; I, hole punch sponge; J, dough-like sponge; K, lobe-like sponge; L, half-and-half sponge; M, tennis ball sponge; N, Myxillina sponge; O, bulb-tipped clump; P, pipe sponge; Q, papilla sponge; R, bubble sponge; S, pancake sponge; T, white dome sponge; U, *Tentorium semisuberites*; V, gray dome sponge; W, volcano sponge; X, slipper sponge; Y, rocket sponge; Z, circle sponge; α, flame sponge. Scale bar  = 20 cm.

**Figure 4:**
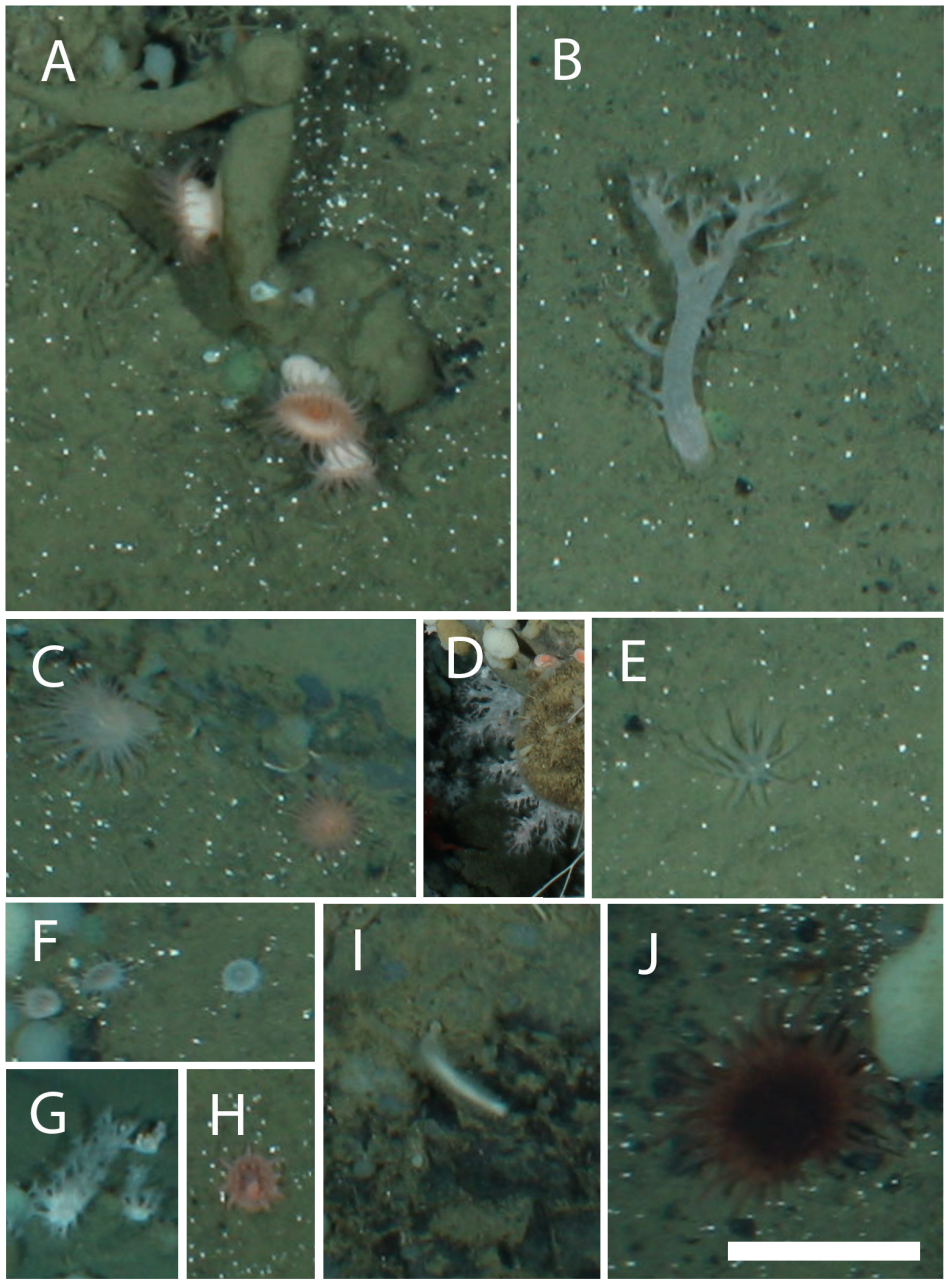
Cnidarians observed at the deep reef. A, Hormathiidae; B, *Gersemia*; C, *Bathyphellia margaritacea*; D, broccoli soft coral; E, large white cerianthid; F, small white actinarian; G, fringe anemone; H, short-tentacled pink anemone; I, sea pen; J, large red anemone. Scale bar  = 5 cm.

**Figure 5:**
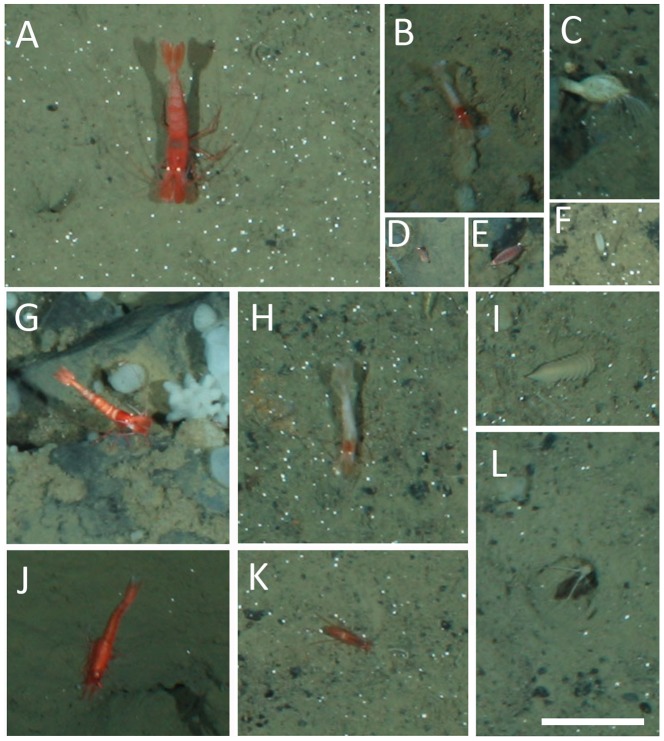
Crustaceans observed at the deep reef. A, *Bythocaris leucopis*; B, small red-and-white shrimp; C, *Verum striolatum*; D, Lysianassidae sp. 1; E, Lysianassidae sp. 2; F, small white isopod; G, dunce hat shrimp; H, fantail shrimp; I, *Saduria megalura*; J, *Birsteiniamysis inermis*; K, *Halirages cainae*; L, *Neohela lamia*. Scale bar  = 5 cm.

**Figure 6:**
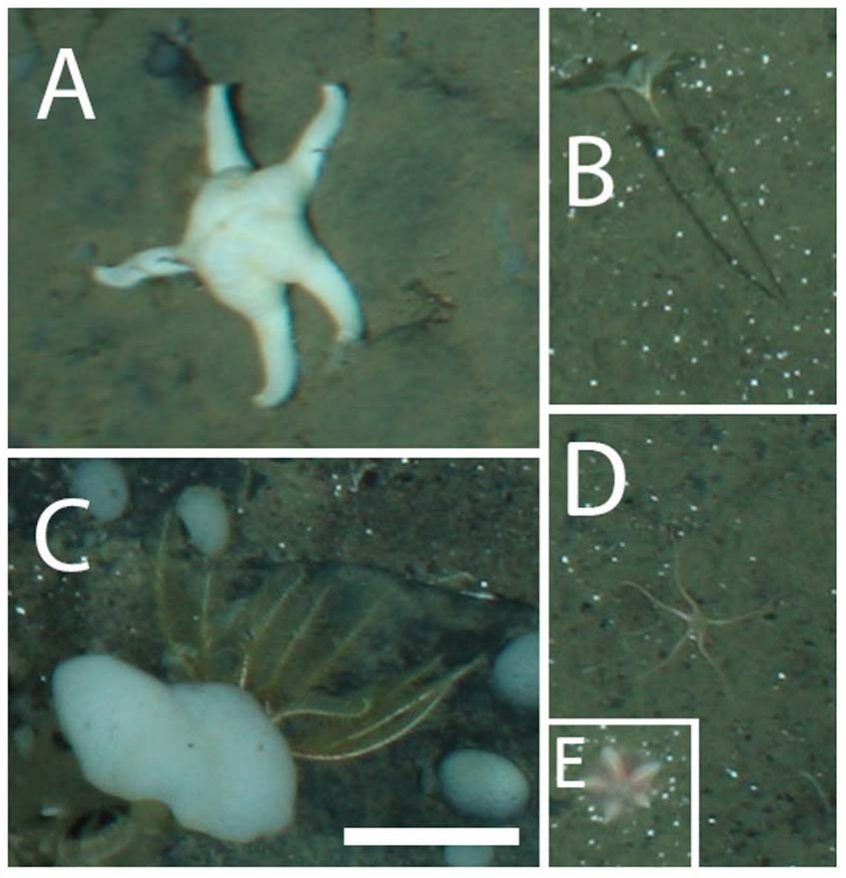
Echinoderms observed at the deep reef. A, *Poraniomorpha hispida*; B, *Bathycrinus carpenterii*; C, *Poliometra prolixa*; D, *Ophiostriatus striatus*; E, *Hymenaster pellucidus*. Scale bar  = 5 cm.

**Figure 7:**
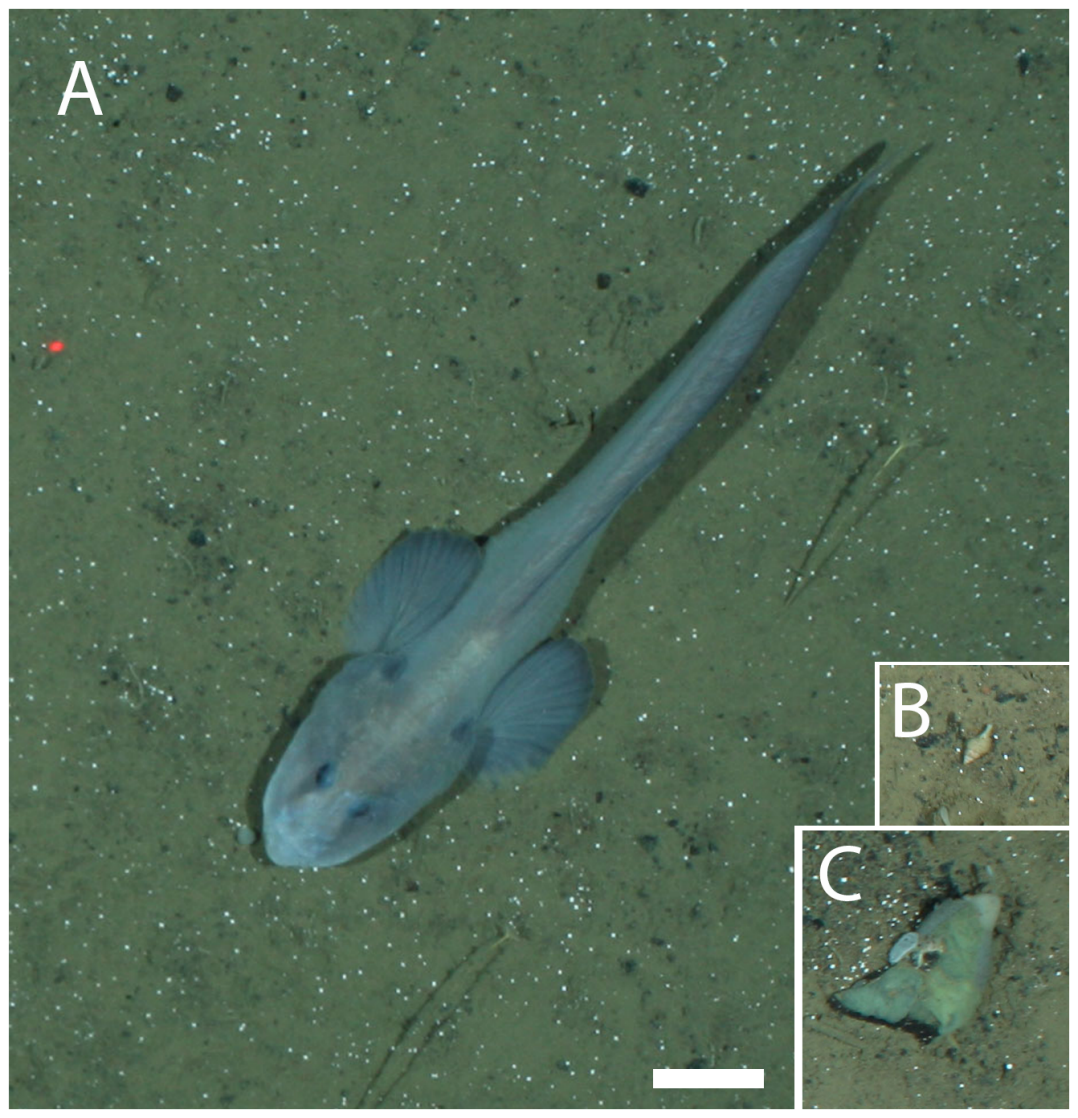
Miscellaneous taxa observed at the deep reef. A, *Lycodes frigidus*; B, *Mohnia mohnia*; C, laminar bryozoan. Scale bar  = 5 cm.

**Figure 8:**
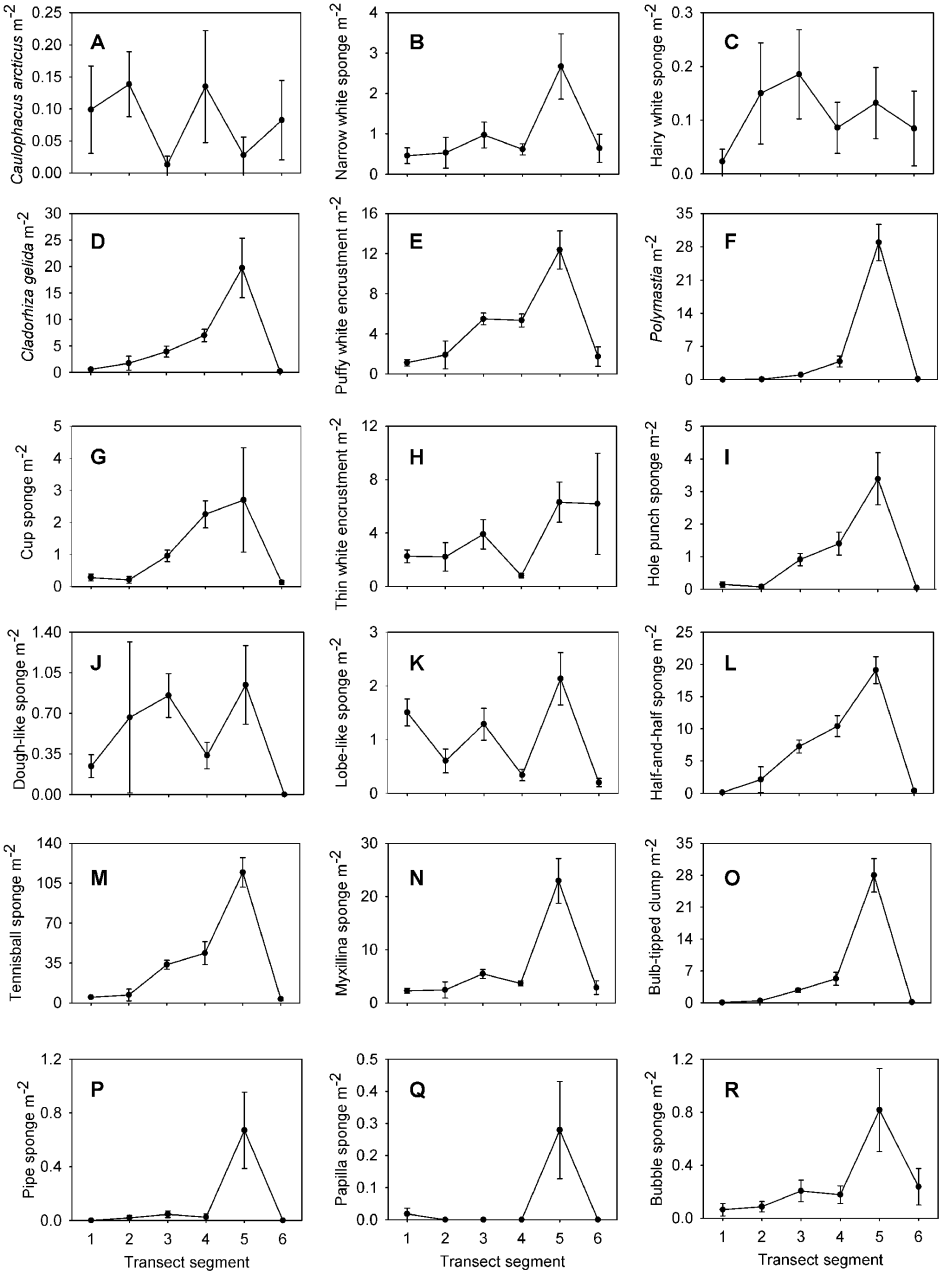
Densities of sponge fauna on each transect segment. Letters as for Fig. 3. Error bars represent standard error. Densities are only shown for taxa which were observed more than once.

**Figure 9:**
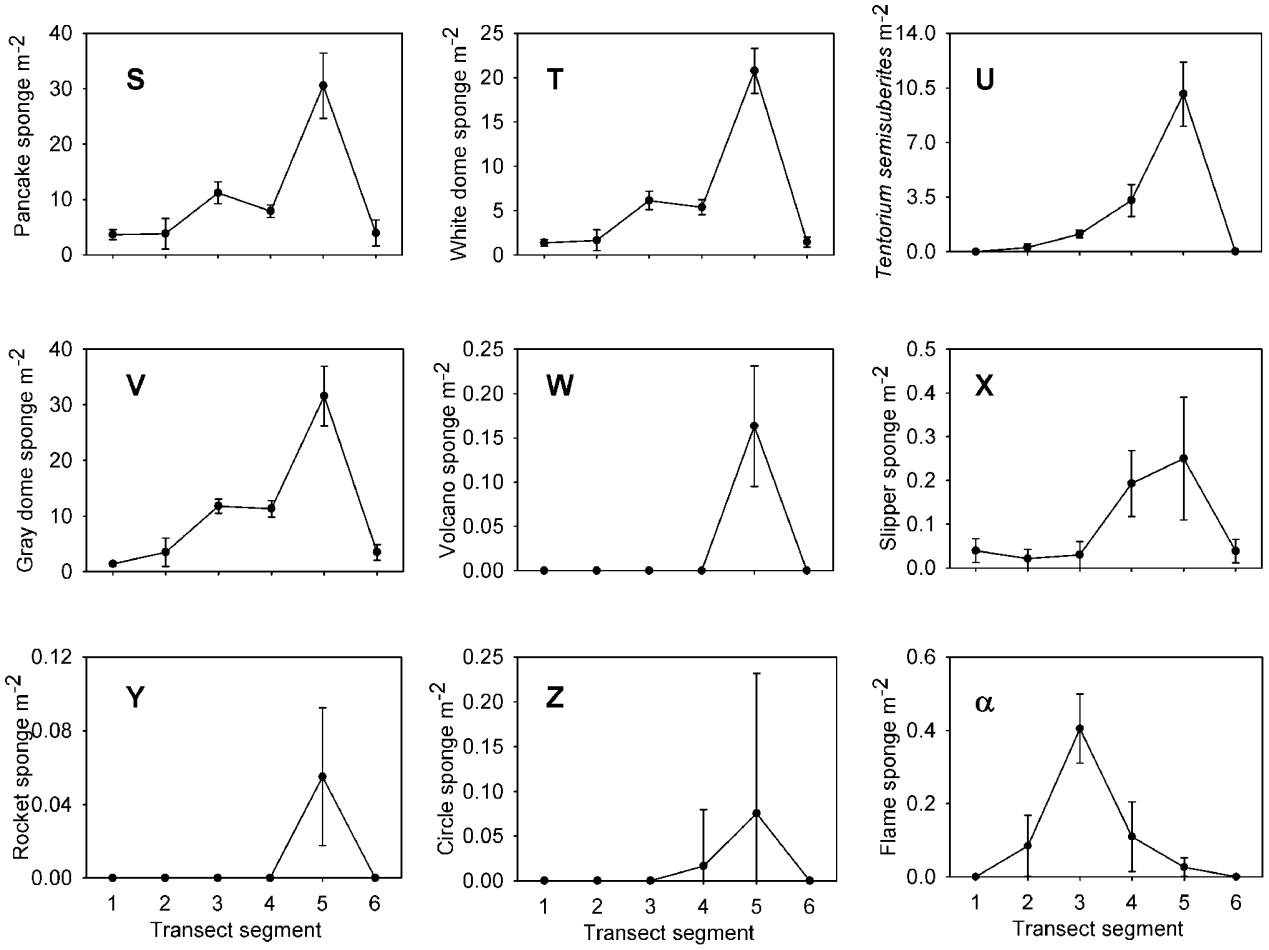
Densities of sponge fauna on each transect segment. Letters as for Fig. 3. Error bars represent standard error. Densities are only shown for taxa which were observed more than once.

**Figure 10:**
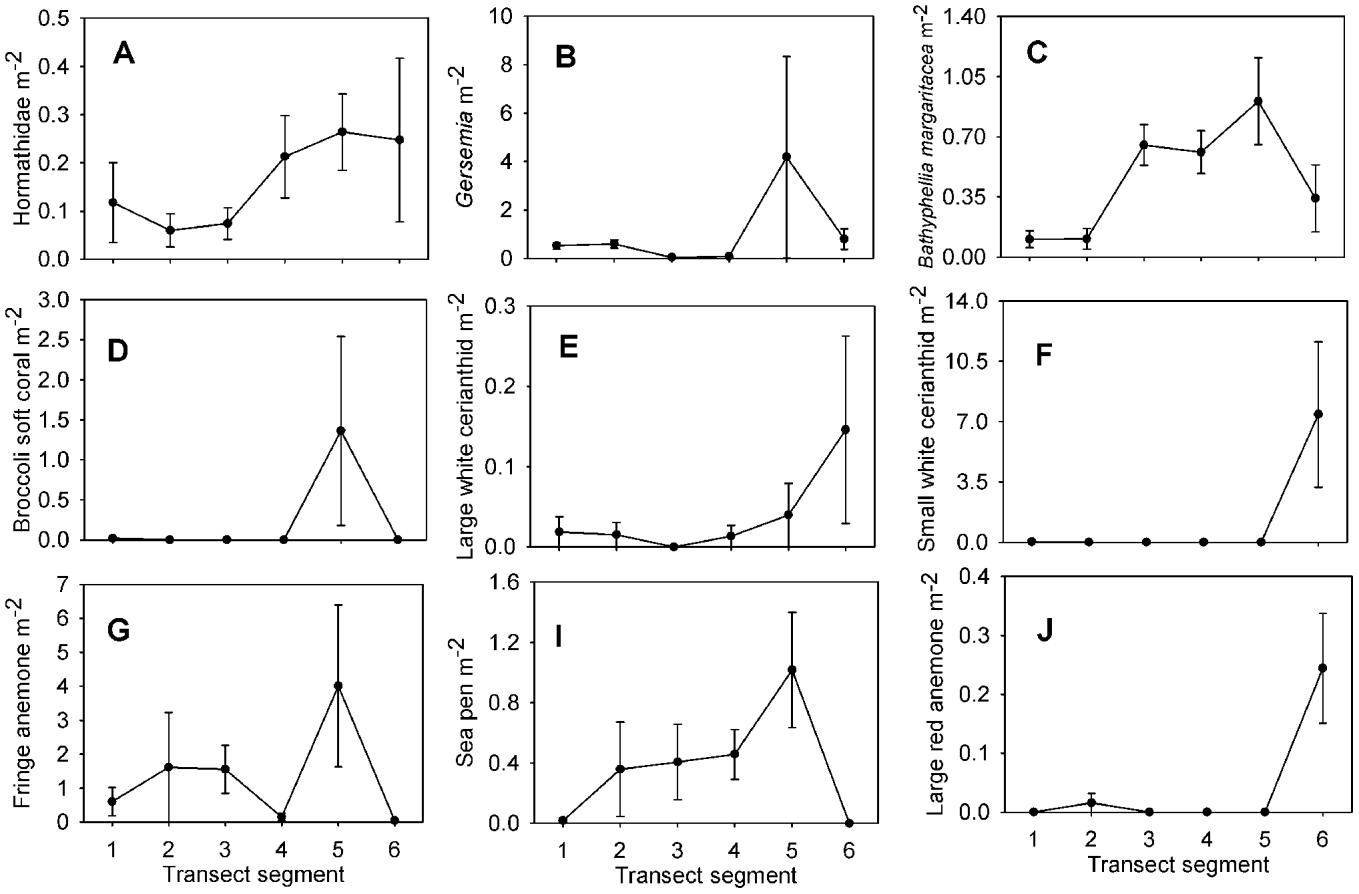
Densities of cnidarian fauna on each transect segment. Letters as for Fig. 4. Error bars represent standard error. Densities are only shown for taxa which were observed more than once.

**Figure 11:**
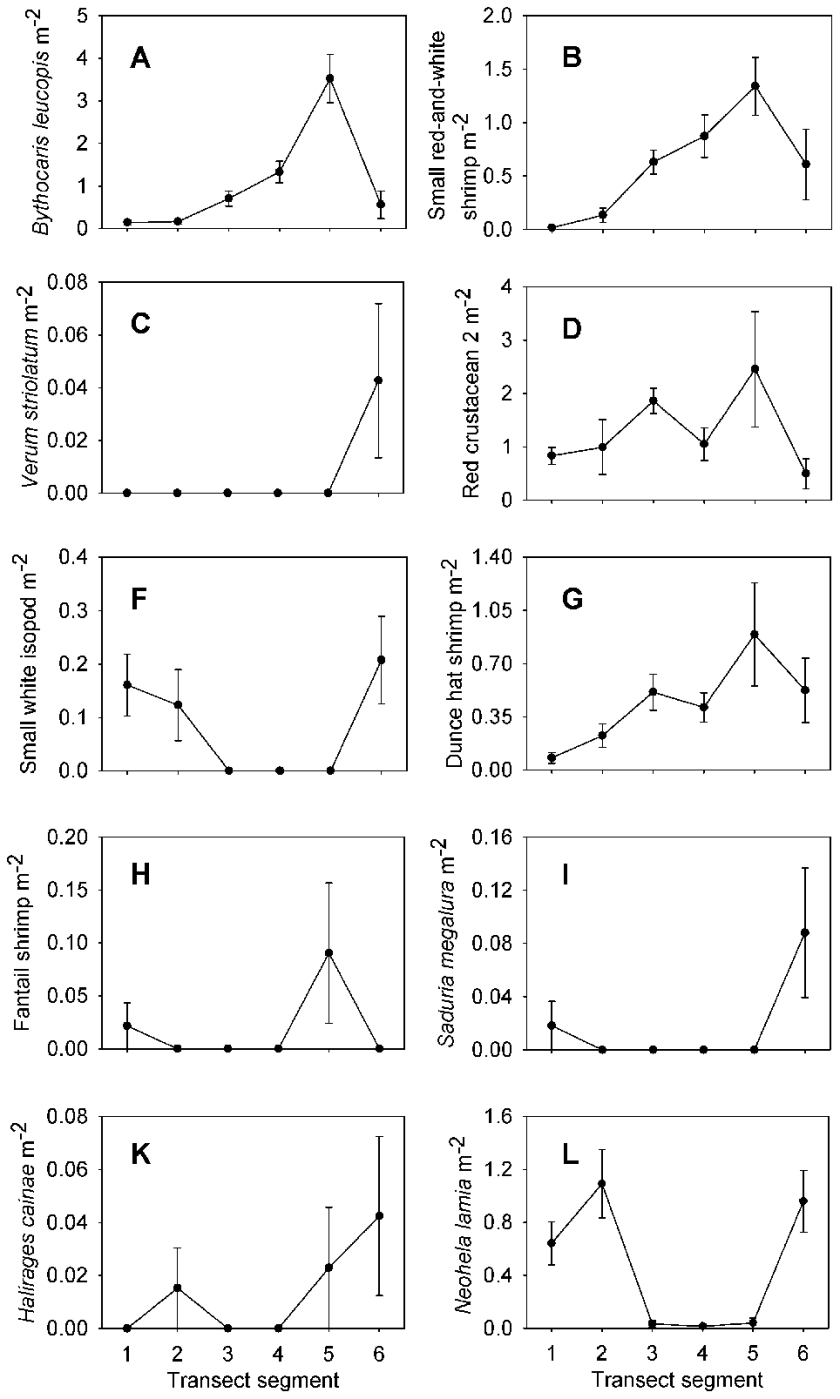
Densities of crustacean fauna on each transect segment. Letters as for Fig. 5. Error bars represent standard error. Densities are only shown for taxa which were observed more than once.

**Figure 12:**
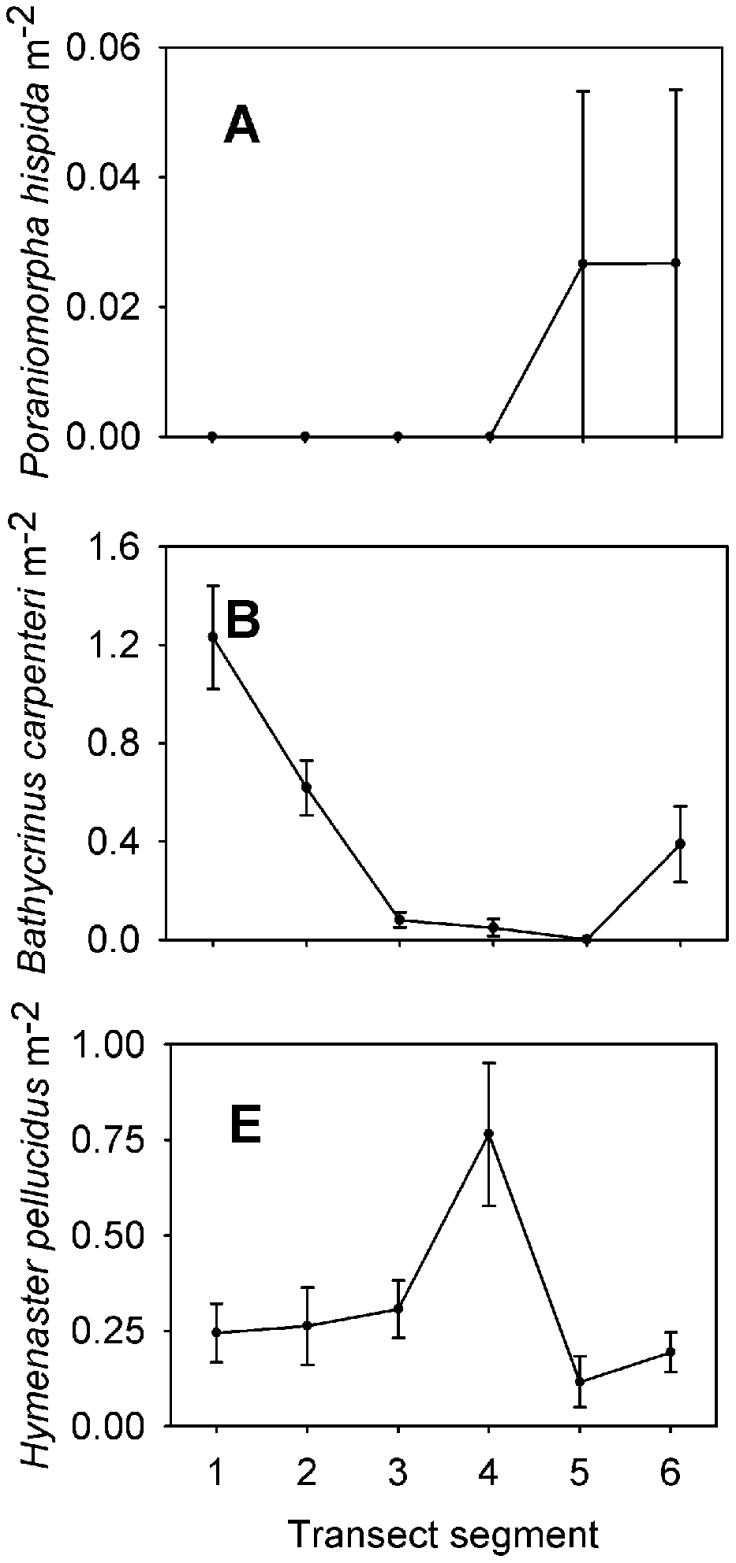
Densities of echinoderm fauna on each transect segment. Letters as for Fig. 6. Error bars represent standard error. Densities are only shown for taxa which were observed more than once.

**Figure 13:**
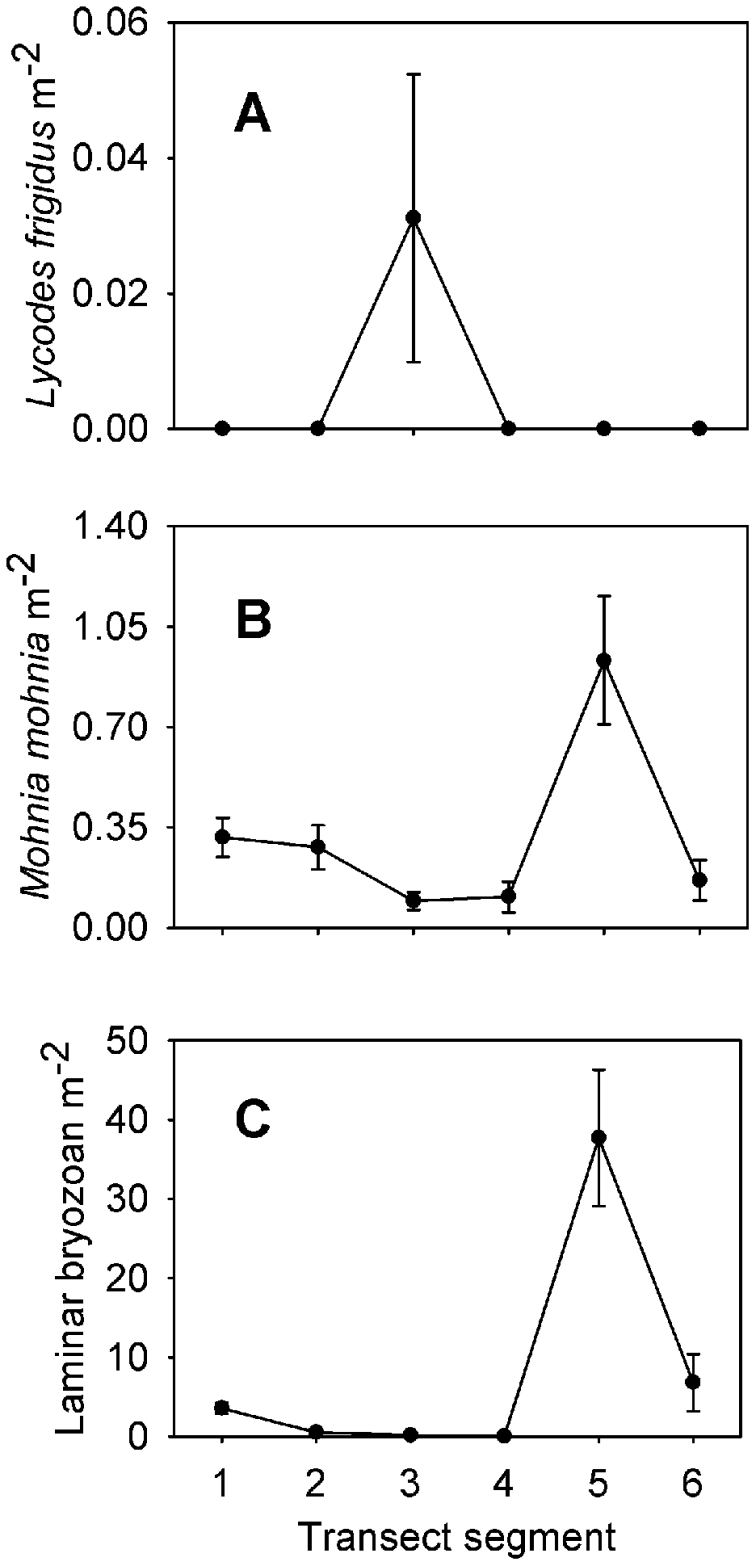
Densities of miscellaneous taxa observed on each transect segment. Letters as for Fig. 7. Error bars represent standard error.

Each taxon is depicted in Figs 3, 4, 5, 6, and 7. Densities of each taxon on each transect segment are shown in Figs 8, 9, 10, 11, 12 and 13. Nineteen taxa had no significant differences in density between transect segments (ANOVA or Kruskal-Wallis test (K-W), p>0.05), and these results are shown in Table S1. In addition, eight taxa showed significant differences in density based on the results of an ANOVA or K-W test (p<0.05); however, post-hoc Bonferroni or Mann-Whitney (M-W) tests failed to show any significant pairwise differences between transect segments. This is likely a by-product of the low α based on the number of pairwise comparisons. Pairwise differences were also sought using the Holm-Bonferroni method [42], but still no pairwise significant differences were revealed. All other taxa showed significant differences in density between at least two transect segments, and the results are listed in Table S1.

Many taxa showed similar patterns of density, with the density of each taxon increasing along the transect and reaching its highest value in transect segment 5, then declining in segment 6. The 17 taxa which fit this pattern are labeled as Group A in Table S1. In addition, some taxa generally increased in density in segments 1–5 but had low density in segment 4. These taxa constitute Group B in Table S1. Taxa which were only present on segment 5 are labeled as Group C in Table S1. Each of the species in Groups A, B, and C had a significant positive correlation to percent hard substratum cover (Spearman correlation, p<0.05; Table S2).

It is worth noting that *Cladorhiza gelida* is present in very high density at the summit of the reef. Indeed in several recorded images, the entire view of the camera (3–4 m^2^) was filled by this “*Cladorhiza* forest.”

In contrast, some taxa could be characterized as soft-sediment fauna, being mostly or exclusively present on segments 1 and 6. These taxa are labeled as Group D in Table S1. An additional group of taxa was also found exclusively on segment 6; these taxa are Group E in Table S1. Taxa in Groups D and E were generally characterized by significant negative correlations to hard-substratum cover, though some showed non-significant correlations (Table S2).

The distribution patterns of a few taxa are unique enough to warrant special mention. Five species, marked as Group F in Table S1, were each present in high density at the summit of the transect (segment 5), and were present elsewhere in significant density only on segments 1 or 6. Each of these species was observed exclusively on hard substrata, and their occurrence on segment 1 was in every case a result of the presence of a dropstone. The hormathiid anemone was in some cases observed on crinoid stalks and *Caulophacus* debris. A similar distribution pattern was observed for the four species in group G, although these taxa did not occur exclusively in the presence of hard substrata.

Both “hairy white sponge” (Fig. 3C) and “flame sponge” (Fig. 3α) reached their highest densities on segment 3, unlike many other species that reached highest density on segment 5. The starfish *Hymenaster pellucidus* was present in its highest density on segment 4. An interesting pattern was observed for “lobe-like sponge” (Fig. 3K) and Lysianassidae sp. 1, both of which were present in higher density on odd-numbered segments (1, 3, and 5) than on even-numbered segments (2, 4, and 6).

### Differences between transect segments

**Figure 14:**
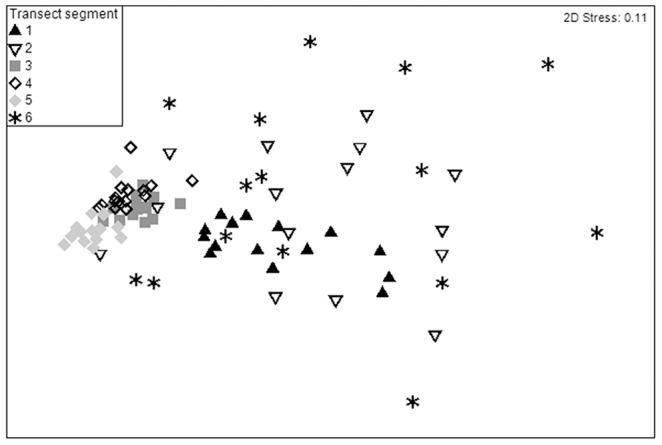
Non-metric multi-dimensional scaling (MDS) plot depicting megafaunal composition on each transect segment. Data have been fourth-root transformed. A 2-D stress value of 0.11 indicates a good fit of the data.

Overall moderate differences in community composition between transect segments were found, which were significant (ANOSIM, Global R = 0.443, p = 0.001). The greatest pairwise differences were between segments 1 and 5 (ANOSIM, R = 0.879, p = 0.001), segments 1 and 4 (ANOSIM, R = 0.806, p = 0.001), segments 3 and 5 (ANOSIM, R = 0.752, p = 0.001), and segments 1 and 3 (ANOSIM, R = 0.730, p = 0.001), respectively. These differences in the taxonomic composition of different transect segments can be visualized in an MDS plot (Fig. 14). Points belonging to segments 3, 4, and 5 form visually coherent groups with points clustered closely both within and between these groups. High within-group similarity for these segments is also shown by the results of the SIMPER routine, which produced within-group similarities of 80.1%, 77.4%, and 77.4%, respectively for segments 3, 4, and 5. The greatest within-group dissimilarity belonged to segment 6, as shown by the widely-dispersed points on the MDS plot and a 36.6% within-group similarity reported by the SIMPER routine. Segments 1 and 2 follow with 62.5% and 45.1% within-group similarity, respectively (SIMPER).

“Tennis ball sponges” (Fig. 3M) were the greatest contributors to within-segment similarity for most transect segments, though the percent contribution was highest (11.2%) for segment 1. The top contributor for segment 2 was *Bathycrinus carpenterii* with 11.6%, and for segment 6 the top contributor was the burrowing amphipod *Neohela lamia* with 12.4% (SIMPER). For segments 3-5, a number of species made minor contributions to within-segment similarity, resulting in no obvious patterns.

**Figure 15:**
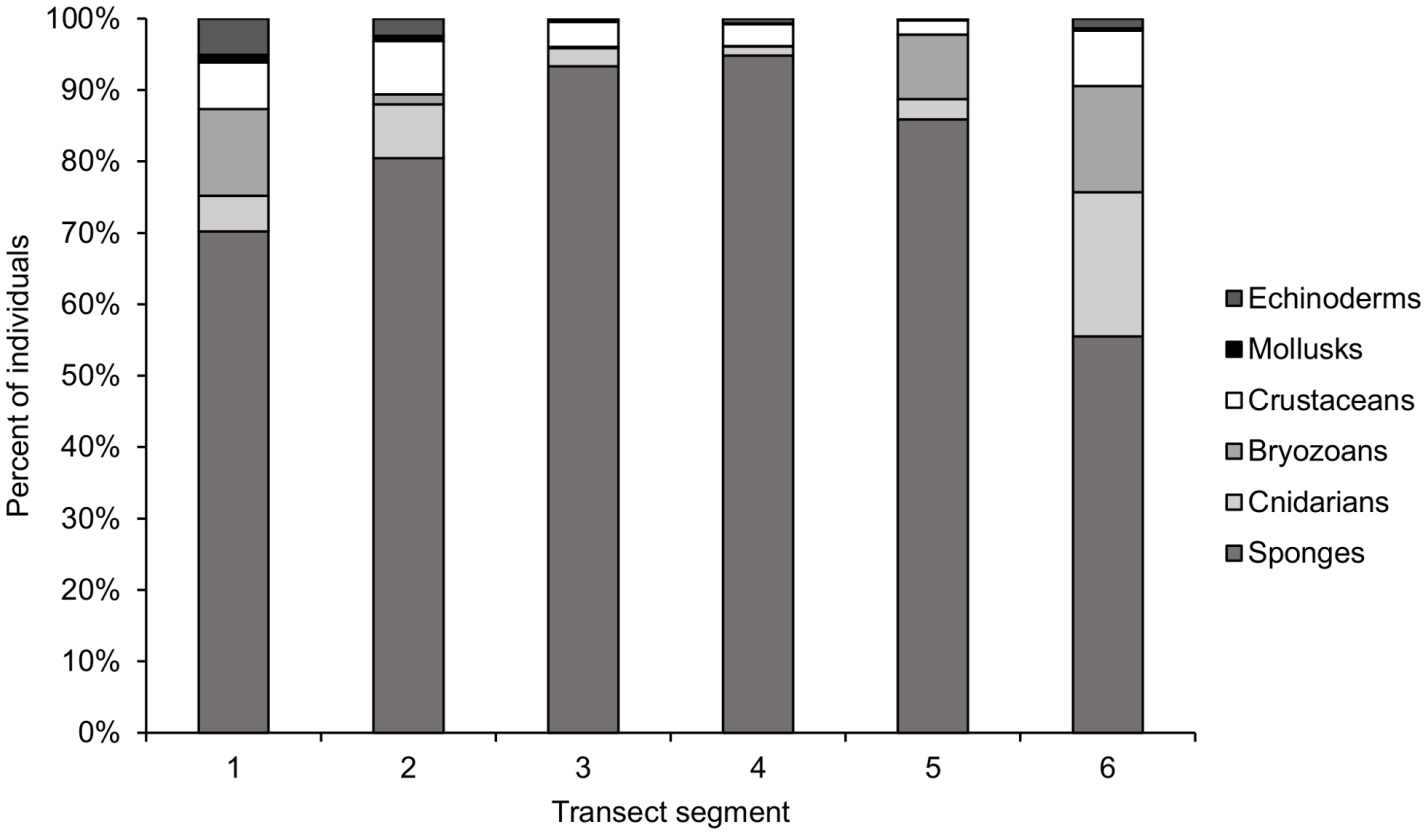
Percent of fauna belonging to each phylum on each transect segment.

Faunal composition throughout the transect is heavily dominated by sponges, the majority of which are likely suspension feeders, especially on hard-substratum segments 3–5 (Fig. 15). The relative proportions of fauna in each phylum are more even on segments 1, 2, and 6, the soft-sediment segments, where predator/scavenger fauna such as crustaceans and echinoderms constitute a larger proportion of the individuals observed (Fig. 15). Cnidarians and bryozoans are also found in higher proportion on segments 1, 2, and 6, though these taxa are most likely suspension feeders (Fig. 15)

**Figure 16:**
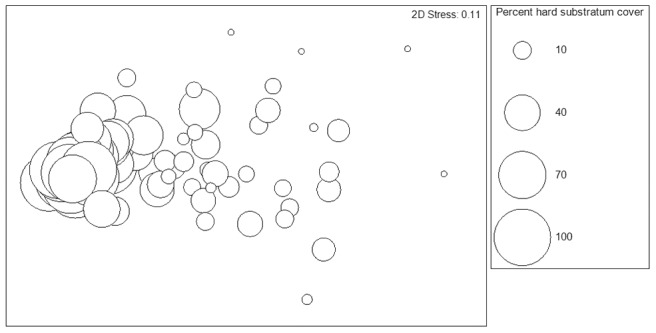
MDS bubble plot depicting megafaunal composition according to percent hard substratum cover. Data have been fourth-root transformed. A 2-D stress value of 0.11 indicates a good fit of the data.

In addition to comparing transect segments, all multivariate analyses were also conducted with respect to hard-substratum cover. It is apparent from the MDS bubble plot (Fig. 16) that images with higher percentage hard substratum cover are more similar to each other. The most widely-dispersed points belong to images with <10% hard substratum cover; those with high (>70%) hard substratum cover form a tight group. This visual result was supported by the SIMPER routine, which reported the highest within-group similarity for images with 60–80% hard-substratum cover (79.3% within-group similarity) and >80% hard-substratum cover (76.7% within-group similarity). The category with the lowest within-group similarity was <20% hard-substratum cover (45.7% within-group similarity).

### Diversity indices

**Figure 17:**
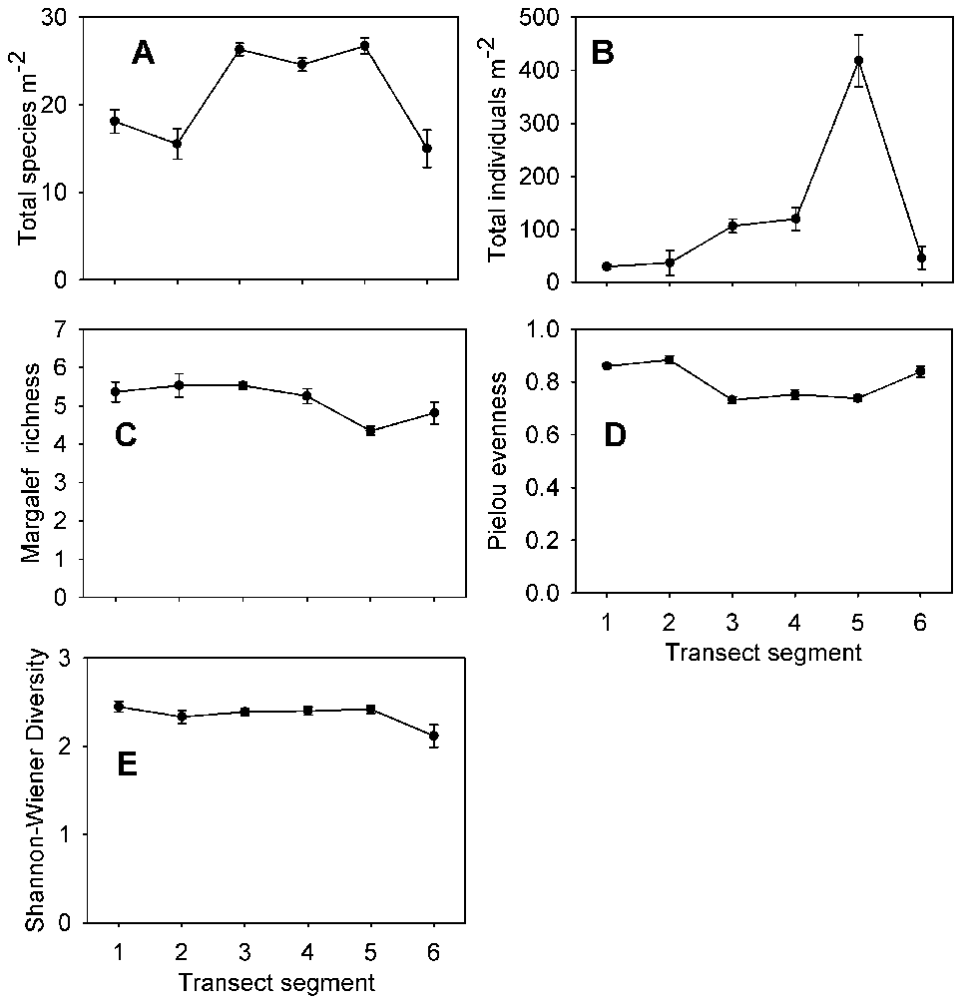
Average of each diversity index on each transect segment. A, total species m^−2^; B, total individuals m^−2^; C, Margalef's richness; D, Pielou's evenness; E, Shannon-Wiener diversity. Error bars represent standard error.

The total density of species was higher on segments with higher percent hard substratum cover (3, 4, and 5) than on segments with primarily soft sediments (1, 2, and 6) (Fig. 17A). Also, the total density of individuals increased between segments 1 and 5, and then dropped again in segment 6 (Fig. 17B). There were significant positive correlations to percent hard-substratum cover for both of these parameters. In contrast, Margalef's richness, which is based on the number of species per number of individuals present, was lowest on segment 5 and not significantly different between the other segments (Table S1, Fig. 17C). Pielou's evenness was significantly higher on the soft-sediment segments (1, 2, and 6) than on segments 3–5 (Fig. 17D). Pielou's evenness was also significantly negatively correlated with hard-substratum cover (Table S2). Shannon-Wiener diversity showed no significant differences between transect segments (K-W, p>0.05; Table S1, Fig. 17E). We assume that the lack of significant differences in this index is due to the opposite trends observed in richness and evenness.

### Biotic habitat features

**Figure 18:**
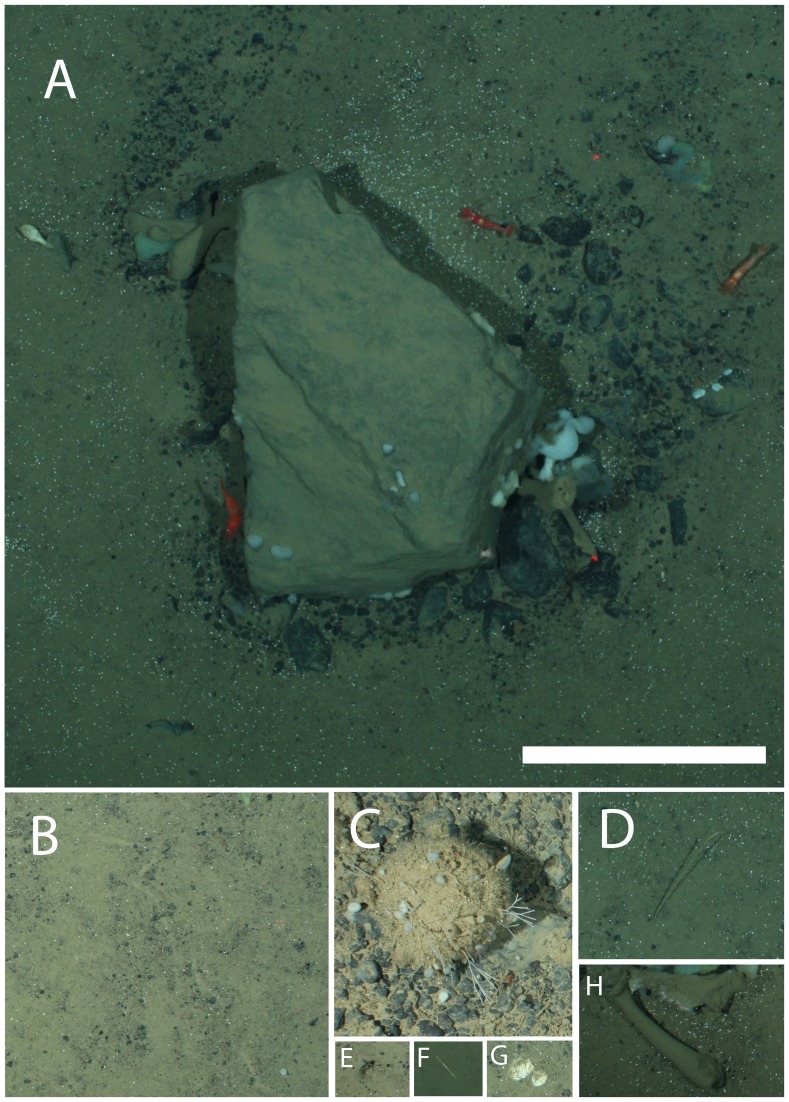
Habitat features observed at the deep reef. A, dropstone; B, lebensspur; C, hairball; D, crinoid stalk; E, burrow entrance; F, worm tube; G, shell fragment; H, *Caulophacus arcticus* debris. Scale bar  = 30 cm.

**Figure 19:**
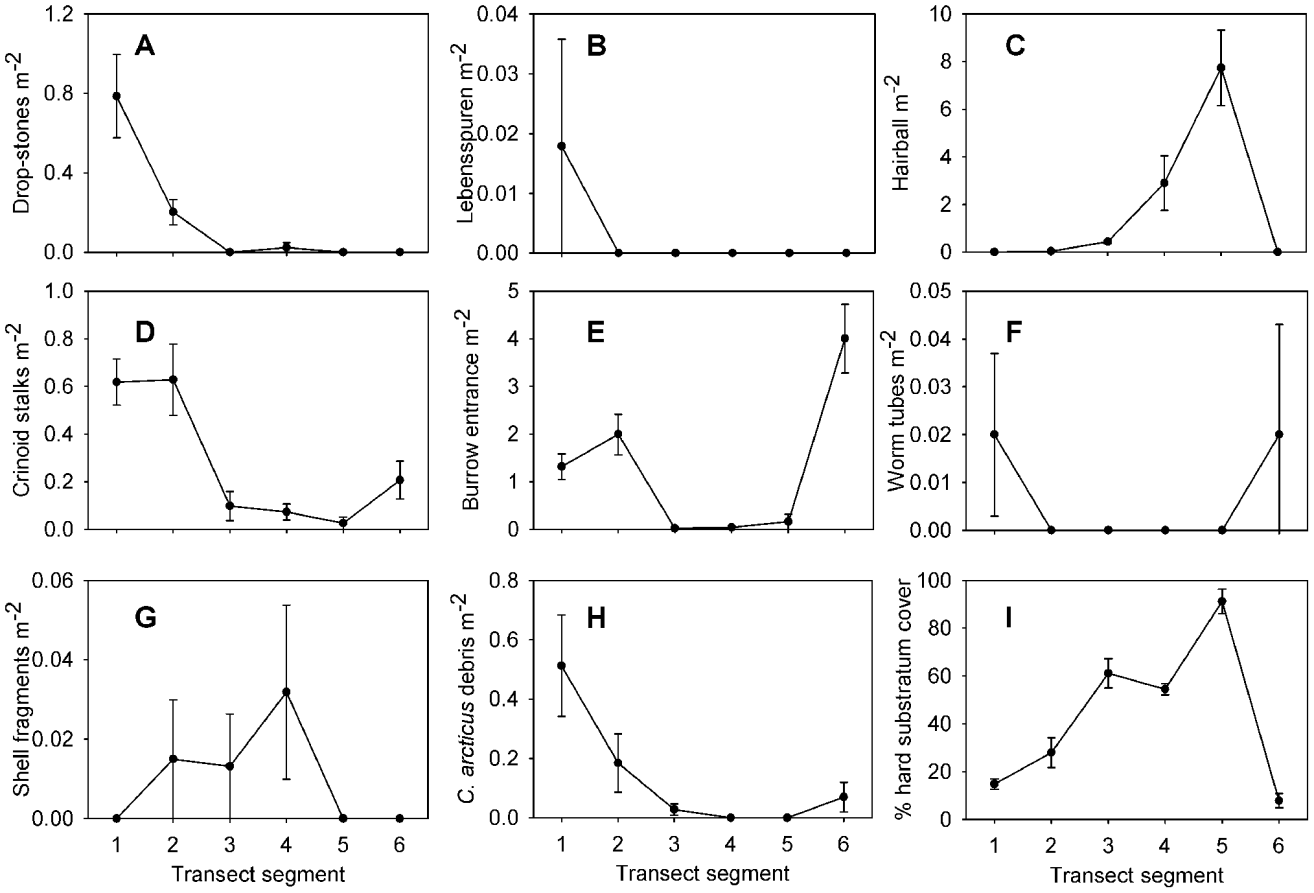
Densities of habitat features observed on each transect segment. Letters as for Fig. 18. I, percent hard substratum cover.

Seven biotic habitat features are depicted in Fig. 18. Of the habitat features, Lebensspuren, shell fragments, and worm tubes showed no significant differences between transect segments, though shell fragments increased in density up to segment 4 (K-W, p>0.05; Table S1, Fig. 19). “Hairballs” increased significantly in density between segments 1 and 5 and showed a significant positive correlation to hard-substratum cover (Table S2). Both crinoid stalks and *C. arcticus* debris occurred in significantly higher density on segment 1, and crinoid stalks were also present in significantly higher density on segment 2 (Table S1, Fig. 19). This pattern most certainly reflects the distribution of each respective species. Densities of each of these structures had a significant negative correlation to hard-substratum cover (Spearman correlation, p>0.05; Table S2). Burrow entrances were also primarily found on soft-sediment segments (segments 1, 2, and 6), having a significant negative correlation to hard-substratum cover (Table S2).

## Discussion

Our results show that suspension feeders, including a wide array of sponges, dominate the community on a steep rocky reef in the deep sea. Previous studies of rocky and coral reefs on the continental shelf have found significant differences between vertical and horizontal rock faces [16], [43], with suspension feeders often dominating on vertical surfaces. Examples include sponges, corals, sea fans, crinoids, brinsingid sea stars, and brachiopods [2], [16], [20], [27], [31], [44], [45]. The dominance of suspension feeders on vertical rock faces and topographic highs is the result of increased food supply as determined by bottom current.

In the present study, the density of suspension feeders is highest on segment 5, where presumably bottom current is fastest, leading to erosion of loose sediment and exposure of bare rock. Segment 5 contains dense stands of *Cladorhiza gelida*, a sponge in the family Cladorhizidae, of which some members are known to be carnivorous [46]. This “*Cladorhiza* forest” is reminiscent of coral or gorgonian stands found on the summits of many seamounts [30], [31], [47] and fjord sills [27]. The high density of *C. gelida* on the summit of the deep-water reef implies high particle/food supplies provided by the swift bottom currents similar to the reason why coral thrives at seamounts [27].

Segments 3 and 4 have comparable species richness to segment 5, but there are significantly lower total densities of individuals on segments 3 and 4. This is probably because lateral food supply is not as high on these segments as on segment 5. Also, several morphospecies of sponges and cnidarians, identified as Group B in Table S1, have lower density on segment 4, indicating their densities are limited by particulate food supply. The carnivorous seastar *Hymenaster pellucidus* [48] reaches its highest density on segment 4, likely because it is not limited by particulate food supply.

Sedimentation is likely to occur on segments 2 and 1 as bottom currents decrease, allowing particles eroded from the reef to settle on the seafloor. We were unable to quantify sedimentation, but organic matter sinking to the seafloor of segment 2 may be an important food source for resident suspension feeders, in particular the stalked crinoid *Bathycrinus carpenterii*, which dominates on this segment.

In shallow water, factors determining the distribution of benthic sessile invertebrates on rocky reefs include light [21], [49], seafloor topography as it affects circulation patterns [15], [27], vertical zonation patterns [20], [44], [50], presence of various water masses [21], [49], herbivory [51] and fish predation [12]. These factors often vary in strength by depth [50]. On the present deep-water reef, factors such as light, herbivory, and surface water masses can obviously be eliminated by virtue of its location in the deep sea. The community of the present deep-water reef is largely influenced by food availability, leading to high densities of sessile species, particularly sponges, at the summit. Even so, the Pielou and Margalef indices indicated that the reef was not as even or species-rich (per individual) as the surrounding abyssal plain community. For segment 5, these patterns can be attributed to the prominence of *Cladorhiza gelida* and tennis ball sponges. It seems the increased food supply on the rocky reef decreases evenness, allowing proliferation and dominance of the species best suited to take advantage of the increased food resource.

The abyssal plain communities above and below the reef are not equivalent, in particular because several species (Group E in Table S1) are present exclusively on segment 6, where the burrowing amphipod *Neohela lamia* is the dominant character species. Segment 1 has higher densities of dropstones and some biotic habitat features such as crinoid stalks, shell fragments, and debris of dead *Caulophacus arcticus*. The availability of such structures enhances habitat heterogeneity on the abyssal plain and may influence community structure [52].

Total faunal densities are higher on segments 1, 2, and 6 than at the nearby slope station HG IV (∼2500 m). There are 18.1, 15.1, and 15.0 individuals m^−2^ on segments 1, 2, and 6 of the present station compared to 12.2, 9.2, and 7.4 individuals m^−2^ at HG IV in 2002, 2004, and 2007, respectively [53]. Additionally, a total of 27 taxa were observed in images from HG IV [53], while 42 taxa were found on each of segments 1, 2, and 6 of the present station. The higher faunal density and number of species on soft-sediment segments of the present station may be the result of food input by sedimentation and the availability of hard substrata. Even on the soft-sediment segments, dropstones occur in higher density on segment 1 (0.8 m^−2^) than at the nearby slope station HG IV (0.3, 0.3, and 0.4 m^−2^ in 2002, 2004, and 2007, respectively [53]). In addition, boulders may potentially break off and roll downhill from the reef, landing on segment 1. Such “outrunner blocks” and dropstones are typically more densely populated than the surrounding sediment [24] and may allow hard-bottom species to persist in an area of predominately soft sediments, such as organisms in Group F. We suspect that the predominantly northwest-flowing bottom current may deliver larvae from the reef summit to outlying dropstones, indicating that these structures may function as “islands” compared to the “mainland” reef.

This study is to the authors' knowledge the first to describe the distribution and diversity of benthic fauna on a rocky reef in the deep sea. By comparing communities found in similar habitats at different depths, we can observe important patterns and factors that influence life in the deep sea.

## Supporting Information

Table S1 **Results of (non-) parametric analyses of variance for all taxa, habitat features, and diversity indices.** K-M, Kruskall-Wallis test; M-W, Mann-Whitney test. For taxa which were only observed once, the transect segment on which the taxon was observed is reported rather than statistical results. Groups are as described in the text.
(DOC)

Table S2 **Results of Spearman correlation to percent hard substratum cover and depth for all taxa, habitat features, and diversity indices.** Correlations with p<0.05 were interpreted as significant.
(DOC)
