# Supplementary material for: High Biodiversity on a Deep-Water Reef in the Eastern Fram Strait
Source: PLoS One. 2014 Aug 25;9(8):e105424. doi: 10.1371/journal.pone.0105424 (PMC4143267; doi:10.1371/journal.pone.0105424)
Supplement: Table S1 — Results of (non-) parametric analyses of variance for all taxa, habitat features, and diversity indices. K-M, Kruskall-Wallis test; M-W, Mann-Whitney test. For taxa which were only observed once, the transect segment on which the taxon was observed is reported rather than statistical results. Groups are as described in the text. (DOC) [file pone.0105424.s001.doc]

| **Taxon** | **Figures** | **Test used** | **f or χ^2^** | **p** | **Significant differences (or location observed)** | **Group** |
| --- | --- | --- | --- | --- | --- | --- |
| *Caulophacus arcticus* | 3A, 8A | K-W | 7.496 | 0.186 | None |  |
| Narrow white sponge | 3B, 8B | K-W | 21.161 | 0.001 | 5>2 | B |
| Hairy white sponge | 3C, 8C | ANOVA | 0.717 | 0.613 | None |  |
| *Cladorhiza gelida* | 3D, 8D | K-W | 50.714 | 0.000 | 3>1, 4>1, 5>1, 3>2, 4>2, 5>2, 3>6, 4>6, 5>6 | A |
| Puffy white encrustment | 3E, 8E | ANOVA log | 23.198 | 0.000 | 3>1, 4>1, 5>1, 3>2, 4>2, 5>2, 3>6, 4>6, 5>6 | A |
| *Polymastia* | 3F, 8F | K-W | 72.800 | 0.000 | 3>1, 4>1, 5>1, 3>2, 4>2, 5>2, 5>3, 5>4, 4>6, 5>6 | A |
| Cup sponge | 3G, 8G | K-W | 40.270 | 0.000 | 4>1, 3>2, 4>2, 5>2, 4>6, 5>6 | A |
| Thin white encrustment | 3H, 8H | K-W | 21.305 | 0.001 | 5>2, 5>4 | B |
| Hole punch sponge | 3I, 8I | K-W | 57.569 | 0.000 | 3>1, 4>1, 5>1, 3>2, 4>2, 5>2, 4>6, 5>6 | A |
| Dough-like sponge | 3J, 8J | K-W | 29.630 | 0.000 | 3>2 | B |
| Lobe-like sponge | 3K, 8K | K-W | 32.472 | 0.000 | 1>4, 1>6, 3>4, 5>4, 5>6 |  |
| Tennisball sponge | 3L, 8L | K-W | 67.036 | 0.000 | 3>1, 4>1, 5>1, 3>2, 4>2, 5>2, 5>3, 5>4, 4>6, 5>6 | A |
| Half-and-half sponge | 3M, 8M | K-W | 68.926 | 0.000 | 3>1, 4>1, 5>1, 3>2, 4>2, 5>2, 5>3, 5>4, 4>6, 5>6 | A |
| Myxillina sponge | 3N, 8N | K-W | 43.926 | 0.000 | 3>1, 5>1, 3>2, 4>2, 5>2, 5>3, 5>4, 5>6 | A |
| Bulb-tipped clump | 3O, 8O | K-W | 72.754 | 0.000 | 3>1, 4>1, 5>1, 3>2, 4>2, 5>2, 5>3, 3>6, 5>4, 4>6, 5>6 | A |
| Pipe sponge | 3P, 8P | K-W | 17.895 | 0.003 | None | C |
| Papilla sponge | 3Q, 8Q | K-W | 16.448 | 0.006 | None | F |
| Bubble sponge | 3R, 8R | K-W | 8.892 | 0.113 | None |  |
| Pancake sponge | 3S, 9S | ANOVA log | 18.837 | 0.000 | 5>1, 3>2, 4>2, 5>2, 3>6, 4>6, 5>6 | B |
| White dome sponge | 3T, 9T | ANOVA log | 37.679 | 0.000 | 3>1, 4>1, 5>1, 3>2, 4>2, 5>2, 5>3, 3>6, 5>4, 4>6, 5>6 | A |
| *Tentorium semisuberites* | 3U, 9U | K-W | 69.513 | 0.000 | 3>1, 4>1, 5>1, 3>2, 4>2, 5>2, 5>3, 3>6, 5>4, 4>6, 5>6 | A |
| Gray dome sponge | 3V, 9V | K-W | 57.737 | 0.000 | 3>1, 4>1, 5>1, 3>2, 4>2, 5>2, 5>3, 5>4, 4>6, 5>6 | A |
| Volcano sponge | 3W, 9W | K-W | 26.149 | 0.000 | None | C |
| Slipper sponge | 3X, 9X | K-W | 9.394 | 0.094 | None |  |
| Rocket sponge | 3Y, 9Y | K-W | 10.112 | 0.072 | None | C |
| Circle sponge | 3Z, 9Z | K-W | 11.587 | 0.041 | None |  |
| Flame sponge | 3α, 9α | K-W | 41.011 | 0.000 | 3>1, 3>2, 3>4, 3>5 |  |
|  |  |  |  |  |  |  |
| Hormathiidae | 4A, 10A | K-W | 8.054 | 0.153 | None | F |
| *Gersemia* | 4B, 10B | K-W | 23.312 | 0.000 | 1>3, 5>1, 2>3 | F |
| *Bathyphellia margaritacea* | 4C, 10C | K-W | 28.094 | 0.000 | 3>1, 4>1, 5>1, 3>2, 4>2, 5>2 | A |
| Broccoli soft coral | 4D, 10D | K-W | 7.251 | 0.203 | None | F |
| Large white cerianthid | 4E, 10E | K-W | 2.267 | 0.811 | None | F |
| Small white actinarian | 4F, 10F | K-W | 19.741 | 0.001 | None | E |
| Fringe anemone | 4G, 10G | K-W | 9.768 | 0.082 | None | B |
| Short-tentacled pink anemone | 4H | ------------- | ----------- | -------- | Segment 1 |  |
| Sea pen | 4I, 10I | K-W | 29.075 | 0.000 | 5>1, 4>6, 5>6 | A |
| Large red anemone | 4J, 10J | K-W | 27.064 | 0.000 | None | E |
|  |  |  |  |  |  |  |
| *Bythocaris leucopis* | 5A, 11A | K-W | 46.255 | 0.000 | 3>1, 4>1, 5>1, 4>2, 5>2, 5>2, 4>6, 5>6 | A |
| Small red-and-white shrimp | 5B, 11B | K-W | 41.584 | 0.000 | 3>1, 4>1, 5>1, 3>2, 4>2, 5>2 | A |
| *Verum striolatum* | 5C, 11C | K-W | 10.112 | 0.072 | None | E |
| Lysianassidae sp. 1 | 5D, 11D | ANOVA log | 4.638 | 0.001 | 3>1, 3>2, 5>6 |  |
| Lysianassidae sp. 2 | 5E | ------------- | --------- | -------- | Segment 4 |  |
| Small white isopod | 5F, 11F | K-W | 20.413 | 0.001 | None | D |
| Dunce hat shrimp | 5G, 11G | K-W | 14.599 | 0.012 | 3>1, 4>1 | A |
| Fantail shrimp | 5H, 11H | K-W | 7.251 | 0.203 | None | G |
| *Saduria megalura* | 5I, 11I | K-W | 11.587 | 0.041 | None | D |
| *Birsteiniamysis inermis* | 5J | ------------- | ---------- | -------- | Segment 6 | E |
| *Halirages cainae* | 5K, 11K | K-W | 5.243 | 0.387 | None | G |
| *Neohela lamia* | 5L, 11L | K-W | 42.892 | 0.000 | 1>3, 1>4, 1>5, 2>3, 2>4, 2>5, 6>4, 6>5 | D |
|  |  |  |  |  |  |  |
| *Poraniomorpha hispida* | 6A, 12A | K-W | 4.046 | 0.543 | None | G |
| *Bathycrinus carpenterii* | 6B, 12B | K-W | 57.377 | 0.000 | 1>3, 1>4, 1>5, 1>6, 2>3, 2>4, 2>5, 6>5 | D |
| *Poliometra prolixa* | 6C | ------------- | ----------- | -------- | Segment 1 |  |
| *Ophiostriatus striatus* | 6D | ------------- | ----------- | -------- | Segment 1 | D |
| *Hymenaster pellucidus* | 6E, 12E | ANOVA | 4.961 | 0.000 | 4>1, 4>2, 4>3, 4>5, 4>6 |  |
|  |  |  |  |  |  |  |
| *Lycodes frigidus* | 7A, 13A | K-W | 10.112 | 0.072 | None |  |
| *Mohnia mohnia* | 7B, 13B | K-W | 26.519 | 0.000 | 5>3, 5>4, 5>6 | G |
| Laminar bryozoan | 7C, 13C | K-W | 53.795 | 0.000 | 1>3, 1>4, 5>2, 5>3, 5>4, 6>4 | F |
|  |  |  |  |  |  |  |
| Dropstone | 18A, 19A | K-W | 46.597 | 0.000 | 1>3, 1>4, 1>5, 1>6, 2>3, 2>5, 2>6 |  |
| Lebensspur | 18B, 19B | K-W | 5.000 | 0.416 | None |  |
| Hairball | 18C, 19C | K-W | 69.865 | 0.000 | 3>1, 4>1, 5>1, 3>2, 4>2, 5>2, 5>3, 3>6, 5>4, 4>6, 5>6 |  |
| Crinoid stalk | 18D, 19D | K-W | 37.441 | 0.000 | 1>3, 1>4, 1>5, 2>3, 2>4, 2>5 |  |
| Burrow entrance | 18E, 19E | K-W | 58.120 | 0.000 | 1>3, 1>4, 1>5, 6>1, 2>3, 2>4, 2>5, 6>3, 6>4, 6>5 |  |
| Worm tube | 18F, 19F | K-W | 4.046 | 0.543 | None |  |
| Shell fragment | 18G, 19G | K-W | 5.243 | 0.387 | None |  |
| *Caulophacus arcticus* debris | 18H, 19H | K-W | 30.032 | 0.000 | 1>3, 1>4, 1>5 |  |
| Percent hard substratum cover | 19I | K-W | 68.100 | 0.000 | 3>1, 4>1, 5>1, 1>6, 3>2, 4>2, 5>2, 2>6, 5>3, 3>6, 5>4, 4>6, 5>6 |  |
|  |  |  |  |  |  |  |
| Total species | 17A | K-W | 41.082 | 0.000 | 3>1, 4>1, 5>1, 3>2, 4>2, 5>2, 3>6, 4>6, 5>6 |  |
| Total individuals | 17B | K-W | 59.252 | 0.000 | 3>1, 4>1, 5>1, 3>2, 4>2, 5>2, 5>3, 3>6, 5>4, 4>6, 5>6 |  |
| Margalef richness | 17C | K-W | 20.032 | 0.001 | 3>5, 4>5 |  |
| Pielou evenness | 17D | K-W | 49.225 | 0.000 | 1>3, 1>4, 1>5, 2>3, 2>4, 2>5, 6>3, 6>5 |  |
| Shannon-Wiener diversity | 17E | K-W | 6.364 | 0.272 | None |  |
