# Supplementary material for: High Biodiversity on a Deep-Water Reef in the Eastern Fram Strait
Source: PLoS One. 2014 Aug 25;9(8):e105424. doi: 10.1371/journal.pone.0105424 (PMC4143267; doi:10.1371/journal.pone.0105424)
Supplement: Table S2 — Results of Spearman correlation to percent hard substratum cover and depth for all taxa, habitat features, and diversity indices. Correlations with p<0.05 were interpreted as significant. (DOC) [file pone.0105424.s002.doc]

| **Taxon**   | **Coefficient** | **p** |  | | --- | --- | --- | | *Caulophacus arcticus* | 0.033 | 0.76 | | Narrow white sponge | 0.591 | 0 | | Hairy white sponge | 0.269 | 0.01 | | *Cladorhiza gelida* | 0.812 | 0 | | Puffy white encrustment | 0.82 | 0 | | *Polymastia* sp. | 0.836 | 0 | | Cup sponge | 0.623 | 0 | | Thin white encrustment | 0.474 | 0 | | Hole punch sponge | 0.716 | 0 | | Dough-like sponge | 0.56 | 0 | | Lobe-like sponge | 0.429 | 0 | | Tennisball sponge | 0.871 | 0 | | Half-and-half sponge | 0.891 | 0 | | Myxillina sponge | 0.708 | 0 | | Bulb-tipped clump | 0.834 | 0 | | Pipe sponge | 0.389 | 0 | | Papilla sponge | 0.223 | 0.035 | | Bubble sponge | 0.437 | 0 | | Pancake sponge | 0.815 | 0 | | White dome sponge | 0.832 | 0 | | *Tentorium semisuberites* | 0.853 | 0 | | Gray dome sponge | 0.824 | 0 | | Volcano sponge | 0.346 | 0.001 | | Slipper sponge | 0.324 | 0.002 | | Rocket sponge | 0.215 | 0.041 | | Circle sponge | 0.285 | 0.006 | | Flame sponge | 0.364 | 0 | |  |  |  | | Hormathiidae | 0.237 | 0.025 | | *Gersemia fruticosa* | -0.37 | 0 | | *Bathyphellia margaritacea* | 0.466 | 0 | | Broccoli soft coral | 0.174 | 0.101 | | Large white cerianthid | -0.064 | 0.546 | | Small white actinarian | -0.23 | 0.029 | | Fringe anemone | 0.32 | 0.002 | | Sea pen | 0.651 | 0 | | Large red anemone | -0.4 | 0 | |  |  |  | | *Bythocaris leucopis* | 0.646 | 0 | | Small red-and-white shrimp | 0.693 | 0 | | *Verum striolatum* | -0.086 | 0.419 | | Lysianassidae sp. 1 | 0.535 | 0 | | Small white isopod | -0.429 | 0 | | Dunce hat shrimp | 0.428 | 0 | | Fantail shrimp | 0.157 | 0.14 | | *Saduria megalura* | -0.296 | 0.005 | | *Halirages cainae* | -0.023 | 0.826 | | *Neohela lamia* | -0.769 | 0 | |  |  |  | | *Poraniomorpha hispida* | 0.034 | 0.748 | | *Bathycrinus carpenterii* | -0.567 | 0 | | *Hymenaster pellucidus* | 0.022 | 0.835 | |  |  |  | | *Lycodes frigidus* | 0.047 | 0.658 | | *Mohnia mohnia* | 0.131 | 0.218 | | Laminar bryozoan | 0.071 | 0.508 | |  |  |  | | Drop-stone | -0.287 | 0.006 | | Lebensspur | -0.1 | 0.347 | | Hairball | 0.777 | 0 | | Crinoid stalk | -0.47 | 0 | | Burrow entrance | -0.863 | 0 | | Worm tube | -0.216 | 0.041 | | Shell fragment | 0.161 | 0.131 | | *Caulophacus arcticus* debris | -0.266 | 0.011 | | Percent hard substratum cover |  |  | |  |  |  | | Total species | 0.795 | 0 | | Total individuals | 0.852 | 0 | | Margalef richness | -0.114 | 0.287 | | Pielou evenness | -0.65 | 0 | | Shannon-Wiener diversity | 0.277 | 0.008 | |  | |  | |
| --- | --- | --- | --- | --- | --- | --- | --- | --- | --- | --- | --- | --- | --- | --- | --- | --- | --- | --- | --- | --- | --- | --- | --- | --- | --- | --- | --- | --- | --- | --- | --- | --- | --- | --- | --- | --- | --- | --- | --- | --- | --- | --- | --- | --- | --- | --- | --- | --- | --- | --- | --- | --- | --- | --- | --- | --- | --- | --- | --- | --- | --- | --- | --- | --- | --- | --- | --- | --- | --- | --- | --- | --- | --- | --- | --- | --- | --- | --- | --- | --- | --- | --- | --- | --- | --- | --- | --- | --- | --- | --- | --- | --- | --- | --- | --- | --- | --- | --- | --- | --- | --- | --- | --- | --- | --- | --- | --- | --- | --- | --- | --- | --- | --- | --- | --- | --- | --- | --- | --- | --- | --- | --- | --- | --- | --- | --- | --- | --- | --- | --- | --- | --- | --- | --- | --- | --- | --- | --- | --- | --- | --- | --- | --- | --- | --- | --- | --- | --- | --- | --- | --- | --- | --- | --- | --- | --- | --- | --- | --- | --- | --- | --- | --- | --- | --- | --- | --- | --- | --- | --- | --- | --- | --- | --- | --- | --- | --- | --- | --- | --- | --- | --- | --- | --- | --- | --- | --- | --- | --- | --- | --- | --- | --- | --- | --- | --- | --- | --- | --- | --- | --- | --- | --- | --- | --- | --- | --- | --- | --- | --- | --- | --- | --- | --- | --- | --- | --- | --- | --- | --- | --- | --- | --- |
|  |  |  |  |  |
